# Identification of copy number variants in whole-genome data using Reference Coverage Profiles

**DOI:** 10.3389/fgene.2015.00045

**Published:** 2015-02-17

**Authors:** Gustavo Glusman, Alissa Severson, Varsha Dhankani, Max Robinson, Terry Farrah, Denise E. Mauldin, Anna B. Stittrich, Seth A. Ament, Jared C. Roach, Mary E. Brunkow, Dale L. Bodian, Joseph G. Vockley, Ilya Shmulevich, John E. Niederhuber, Leroy Hood

**Affiliations:** ^1^Institute for Systems BiologySeattle, WA, USA; ^2^Inova Translational Medicine Institute, Inova Health SystemFalls Church, VA, USA

**Keywords:** whole-genome sequencing, structural variation, depth of coverage, signal processing, clinical genomics

## Abstract

The identification of DNA copy numbers from short-read sequencing data remains a challenge for both technical and algorithmic reasons. The raw data for these analyses are measured in tens to hundreds of gigabytes per genome; transmitting, storing, and analyzing such large files is cumbersome, particularly for methods that analyze several samples simultaneously. We developed a very efficient representation of depth of coverage (150–1000× compression) that enables such analyses. Current methods for analyzing variants in whole-genome sequencing (WGS) data frequently miss copy number variants (CNVs), particularly hemizygous deletions in the 1–100 kb range. To fill this gap, we developed a method to identify CNVs in individual genomes, based on comparison to joint profiles pre-computed from a large set of genomes. We analyzed depth of coverage in over 6000 high quality (>40×) genomes. The depth of coverage has strong sequence-specific fluctuations only partially explained by global parameters like %GC. To account for these fluctuations, we constructed multi-genome profiles representing the observed or inferred diploid depth of coverage at each position along the genome. These Reference Coverage Profiles (RCPs) take into account the diverse technologies and pipeline versions used. Normalization of the scaled coverage to the RCP followed by hidden Markov model (HMM) segmentation enables efficient detection of CNVs and large deletions in individual genomes. Use of pre-computed multi-genome coverage profiles improves our ability to analyze each individual genome. We make available RCPs and tools for performing these analyses on personal genomes. We expect the increased sensitivity and specificity for individual genome analysis to be critical for achieving clinical-grade genome interpretation.

## Introduction

Deletions, duplications and other copy number variations (CNVs) are important components of genomic structural variation (SV), which need to be assessed when studying individual genomes in a personal or clinical context. Accurate identification of DNA copy numbers from short-read sequencing data remains a challenge (Teo et al., 2012) for a variety of reasons, including the voluminous file sizes, the short-read lengths, and insert sizes relative to the length of interspersed repeats, sequence-specific biases, and the lack of quality control standards.

Many tools have been developed for detecting CNVs from “second generation” short-read re-sequencing data, based on one or more of four signal detection methods: (1) read pair or paired end mapping (Chen et al., 2009; Korbel et al., 2009; Quinlan et al., 2010; Chiara et al., 2012; Krishnan et al., 2012; Marschall et al., 2012; Yasuda et al., 2012), (2) split-read mapping (Ye et al., 2009; Wang et al., 2011; Zhang et al., 2011; Emde et al., 2012; Karakoc et al., 2012; Schröder et al., 2014), (3) read depth analysis (Chiang et al., 2009; Xie and Tammi, 2009; Yoon et al., 2009; Ivakhno et al., 2010; Zhang et al., 2010; Abyzov et al., 2011; Magi et al., 2011; Miller et al., 2011; Xi et al., 2011; Klambauer et al., 2012; Szatkiewicz et al., 2013; Wang et al., 2013; Nguyen et al., 2014), and (4) *de novo* assembly (Nijkamp et al., 2012; Chen et al., 2014; Rizk et al., 2014). Assembly approaches, however, tend to function as verification methods rather than discovery tools.

Read-pair algorithms consider discordant pairs of reads, or pairs that diverge from the expected size or orientation. They then cluster these reads into independent events and apply quality filters. The methods differ mostly in how they cluster discordant reads, but also in the filtering steps. In an effort to improve sensitivity, some methods also include ambiguously mapped reads. Called soft clustering, these approaches assign the ambiguous reads to a mapping that then clusters with an event. Tools that employ this method include HYDRA (Quinlan et al., 2010), VariationHunter (Hormozdiari et al., 2010), and GASVPro (Sindi et al., 2012). A few tools, such as ChopSticks (Yasuda et al., 2012) and CLEVER (Marschall et al., 2012) also consider concordant reads in order to refine breakpoint locations.

Split-read mapping detects breakpoints by aligning different portions of a read to separate locations in the reference genome. This approach is computationally taxing, so different methods use different heuristics to guide read alignment. MATCHCLIP (Wu et al., 2013) studies CIGAR strings (Li et al., 2009) to find reads with long soft clipped segments that overlap. The Pindel tool (Ye et al., 2009) looks for paired reads for which one read did not align to the reference, then searches nearby for split read mapping of the unaligned read. CREST (Wang et al., 2011) uses multiply aligned reads with soft clips, gaps inserted at the end of the read when matching to the reference is low, to help guide mapping. SplazerS (Emde et al., 2012) defined its own mapping strategy, which does not depend on heuristics, and while its results are quite sensitive the runtimes are large. Split-read methods are sensitive, especially to shorter events, but they are limited by coverage, length of reads, runtimes, and by the presence of interspersed repeats at the boundaries of CNVs. Such methods may be best suited for small genomes and non-complex regions of the human genome.

Methods based on read depth (depth of coverage) largely differ by the statistical model they use to detect CNVs. CNVeM (Wang et al., 2013) takes advantage of maximum likelihood estimations to determine copy number, GENSENG (Szatkiewicz et al., 2013) employs a hidden Markov model (HMM), and CNVnator (Abyzov et al., 2011) uses a mean-shift approach in modeling the data. Some methods analyze multiple samples at once to more accurately model the coverage across a given region (Zhang et al., 2010; Magi et al., 2011; Klambauer et al., 2012; Nguyen et al., 2014). Similarly, these methods differ by the statistical method they use, for example cn.MOPS (Klambauer et al., 2012) employs a mixed Poisson model while CNVrd2 (Nguyen et al., 2014) uses a normal (Gaussian) mixture model. The strength of read depth methods is their ability to detect large CNVs. However these methods are typically limited in their ability to detect smaller events and have poor breakpoint resolution, as compared to the other approaches.

Some tools integrate multiple signals in order to increase accuracy, and can be divided into three general strategies. One strategy uses a primary signal to generate candidate CNV calls, then refine or support those calls with a secondary signal, most commonly pairing read depth and read pair methods (Medvedev et al., 2010; Handsaker et al., 2011; Qi and Zhao, 2011; Zhang and Wu, 2011; Bellos et al., 2012; Jiang et al., 2012; Rausch et al., 2012; Sindi et al., 2012; Zhu et al., 2012; Escaramís et al., 2013; Hart et al., 2013; Mimori et al., 2013). A second strategy runs multiple signal detection methods independently, then merges the results together (Wong et al., 2010; Lam et al., 2012). Finally, the third strategy integrates multiple signal types into a statistical model to generate combined CNV calls (Shen et al., 2011; Hayes et al., 2012; Michaelson and Sebat, 2012; Layer et al., 2014).

While whole-genome sequencing (WGS) providers target a global metric of depth of coverage—e.g., 40-fold for high-quality genomes—the depth of coverage has both statistical and strong sequence-specific fluctuations. These fluctuations are only partially explained by global parameters like %GC, and pose a significant deconvolution problem. At the extreme of low coverage, “dropout” regions lack sufficient coverage to determine the individual's genotype reliably. In addition to centromeres, heterochromatin, and other gaps in the reference sequence, a fraction of the genome is not observed due to random fluctuations in read distribution, or due to technology biases.

Normalizing the local depth of coverage to the average global depth of coverage may lead to large numbers of false-positive CNV identifications. A further complication arises from the fact that interspersed repeats mediate many genome rearrangements and are thus frequently observed at the boundaries of CNVs and other SVs. As a result, short sequence reads at the boundaries of such events may be particularly difficult to map. Many analysis methods working on individual genomes thus frequently misidentify structural variants (SVs), particularly hemizygous deletions in the 1–100 kb range.

The depth of coverage along the genome can be modeled by comparing coverage profiles across samples, e.g., in cn.MOPS (Klambauer et al., 2012). A significant limitation of this approach is the requirement for several genomes for joint analysis (e.g., at least six in cn.MOPS)—a requirement that may pose challenges in a clinical context. Even for a single genome, managing the coverage data is a hurdle due to the multi-gigabyte file sizes involved.

We present here (1) a very efficient compressed format for storing coverage information, and (2) a method for identification of CNVs, based on coverage normalization to pre-computed profiles derived from a large cohort of genomes. Our method simplifies the management of depth of coverage data and enables efficient analysis of individual genomes.

## Materials and methods

### Description of data set

We have analyzed depth of coverage in 6392 human whole-genome assemblies from 6135 individuals, most in trios or larger families. None of these genomes are derived from cancer samples or cell lines. Some of these genomes (*n* = 199) were assembled using more than one pipeline version; we consider the most recent assembly for a genome to be “primary,” and older assemblies “non-primary.” Also, 194 genomes were sequenced on both the Complete Genomics, Inc. (CGI) and Illumina platforms. The genomes were sequenced at high quality (>40× average coverage). The components of the data set are detailed as follows.

- ISB-CGI: a set of 1308 primary genome assemblies sequenced by CGI for the Institute for Systems Biology (ISB);
- ITMI-CGI: a set of 2439 primary genome assemblies sequenced by CGI for the Inova Translational Medicine Institute (ITMI) (Bodian et al., 2014);
- Diversity-CGI: a set of 69 genomes publicly released by CGI (http://www.completegenomics.com/public-data/69-Genomes/);
- ITMI-Illumina: a set of 2456 primary genome assemblies sequenced by Illumina for ITMI.
- The Combined-CGI set includes the 3816 primary genome assemblies in sets #1, #2, and #3.

The ISB-CGI genomes were produced and analyzed using a variety of library construction and analytic pipeline versions (Supplementary Figure 1), as follows:

- CGI library v.1: 1173 assemblies (1026 primary); CGI library v.2: 286 assemblies (282 primary).
- CGI pipeline software versions 1.08.0.30 through 1.08.0.34: 66 assemblies (61 primary).
- Pipeline versions 1.10.0.22 through 1.12.0.47: 137 assemblies (106 primary).
- Pipeline versions 2.0.1.6 through 2.4.0.43: 870 assemblies (859 primary).
- The 286 library v.2 assemblies were processed with pipeline versions 2.5.0.19 and 2.5.0.20 (282 primary).

The 2439 ITMI-CGI assemblies were all produced using CGI library v.1 and pipeline versions 2.0.0.37 through 2.0.4.18 (all primary). The Diversity-CGI assemblies were produced with pipeline versions 1.10.0.2 through 1.10.0.26 and then reassembled using pipeline version 2.0.0.26. The NA12878 genome was re-sequenced by CGI and analyzed with pipeline version 2.5. The ITMI-Illumina genomes were processed using versions 2.0.0 through 2.0.2 of Illumina's standard genome analysis pipeline.

### Preprocessing of genome coverage

For genomes sequenced on the CGI platform, we obtain per-base depth of coverage information from the coverage report in the “REF” directory, using the “gcCorrectedCoverage” column. This column was added to the report in version 1.10 of the pipeline; we therefore used instead the “weightSumSequenceCoverage” column for assemblies computed on earlier pipeline versions. For genomes sequenced on the Illumina platform, we extracted the per-base coverage profile from BAM files using samtools depth (Li et al., 2009). For efficient storage and analysis, we transformed each genome's coverage report into a compact binary format. In this format, one byte is used to represent the average coverage values for each non-overlapping, 20 bp window. Since the average coverage may exceed the maximal value that can be represented with one byte, we implemented a minimally lossy representation format with three representation regimes (Supplementary Figure 2), as follows. Coverage up to 200-fold is represented unmodified. Coverage above 200 and under 2700—a small fraction of the genome—is transformed using the formula int(sqrt(coverage–200)+200). Coverage above 2700 is stored in a separate file and at full resolution (i.e., not binned); such “overflow” sites, typically present in the mitochondrial chromosome and in very high copy-number segments, are rare and of special interest. The resulting binary format (which is identical for both technologies) is then indexed using tabix (Li, 2011) for efficient retrieval of coverage data.

### Genome stratification by %GC

The %GC of a sequence is known to affect its depth of coverage (Rieber et al., 2013). Sequences of extreme %GC have lower complexity than sequences of intermediate %GC, which makes unique mapping of reads more difficult. Sequencing technologies may also behave differently on sequences with different %GC due to biochemical differences in the sequenced DNA. Furthermore, the relative coverage over different %GC levels may vary between batches of samples analyzed at different times.

To control for such biases, we stratified the genome at 1-kb resolution into 25 %GC “buckets,” each having approximately equal total genomic span (1/25 of the genome). We thus bin the genome by rank instead of by equally spaced %GC cutoffs, to avoid corrections based on bins that hold too little data (i.e., extreme %GC). For the GRCh37 (hg19) freeze of the human genome, the cutoffs used to separate between these %GC ranges were: 30.1, 31.7, 32.9, 33.9, 34.8, 35.6, 36.3, 37.0, 37.7, 38.3, 39.8, 39.6, 40.3, 41.0, 41.8, 42.6, 43.4, 44.4, 45.4, 46.5, 47.9, 49.5, 51.9, and 56.0%.

### Scaling of coverage signal

Since individual genomes may be sequenced to different total depths, the comparison of coverage values across samples necessitates normalization of read depth for each sample to a common scale. The simplest method involves scaling the depth of each genome to the total coverage, in similarity to the scaling of transcriptome samples to their total counts (Meyers et al., 2004). We implemented a more nuanced scaling approach borrowing concepts from our digital transcriptome normalization methods (Glusman et al., 2013). To avoid sex-specific coverage biases and variable mitochondrial representation, we consider only sequence coverage in autosomes. We further excluded “overflow” coverage sites typically observed in high copy number segments. Finally, we computed the total coverage for scaling separately for each of the 25 %GC buckets. Thus, each genome is characterized by a “characteristic coverage vector” of 25 values representing the total autosomal coverage in %GC buckets, excluding “overflow” sites. The characteristic coverage vector serves as a fingerprint for comparing genomes and for optimizing scaling factors.

For a set of genomes sharing some characteristic, such as a sequencing technology or pipeline version, we compute a 25-value “target coverage vector” as the geometric average of the corresponding characteristic coverage vectors of the studied genomes. The target coverage vector is a characteristic of a set of genomes, and is computed only once per set. Finally, the depth of coverage along each chromosome in a genome is equalized to a common scale by dividing by the target coverage value for the corresponding %GC bucket.

### Generation of reference coverage profiles

The expected ploidy of the genome varies; on autosomes, the X chromosome in females, and the pseudo-autosomal regions (PARs) in males, the genome is expected to be diploid; in males, the sex chromosomes outside PARs are expected to be haploid. We estimate a Reference Coverage Profile (RCP): the scaled coverage level corresponding to diploid coverage (regardless of expected ploidy), in each 1-kb segment of the genome. For most of the genome, the median coverage serves as an excellent and simple estimate of the diploid level. Where deletions and duplications are common in the population, though, correct estimation of the diploid level necessitated applying the following three heuristics. First, across a set of genomes, the scaled coverage level should cluster near integer multiples of the haploid coverage level. Second, the most abundant cluster (peak) should represent the expected ploidy; when the expected ploidy is diploid, additional peaks may represent one copy (hemizygosity), zero copies (nullizygosity), or other, higher copy number variants (CNVs) (Supplementary Figure 3). To avoid trivial solutions, we further penalize solutions that yield genotype distributions that deviate from Hardy–Weinberg equilibrium.

At 1-kb resolution, this yields a very compact representation (<10 MB) of the empirically observed coverage levels along the genome. We computed separate RCPs for the two technologies (CGI and Illumina) and for 10 version ranges of CGI's analytic pipeline (Supplementary Figure 1), including up to 500 genome assemblies per RCP. The diploid coverage level, which we estimated empirically from collections of genomes, cannot be explained or predicted from the %GC and the mapability of the sequence (Supplementary Figure 4).

### Normalization of coverage to the reference value

Given an individual genome's scaled coverage profile, we divide the scaled coverage in each kb-sized bin by the corresponding reference value to obtain the genome's normalized coverage profile (NCP). Normalized values near 1 represent the expected diploid coverage, values near 0.5 represent hemizygosity (including chromosomes X and Y in males), and values near 0 represent nullizygosity. Conversely, values larger than 1 may represent duplications and higher-count CNVs.

A typical file size for a genome's normalized coverage expressed at 1-kb resolution is 8.6–10 MB. This representation is small enough to support incorporating coverage analysis into routine genome analysis pipelines.

### Segmentation of normalized coverage

After normalizing each genome's coverage to the corresponding RCP, we sought to identify deletions and higher copy number segments. To achieve this, we segmented the NCP using HMMSeg (Day et al., 2007), a program for segmentation of continuous genomic data using HMMs.

We created an HMM with five states, representing the number of observed copies in a locus: state 0 represents nullizygosity (no coverage or complete deletion), state 1 typically represents hemizygosity (one copy only), state 2 corresponds to normal diploid zygosity, state 3 denotes observation of an extra copy, and state 4 represents observing four or more copies. Each state is associated with an emission value in terms of normalized coverage (0, 50, 100, 150, and 200%, respectively). The model parameters include, for each state, the allowed variance of emission and the transition probabilities to each state (Supplementary Figure 5). HMMSeg computes for each bin the most probable state; we then segment the genome by identifying consecutive bins with the same state.

### Computation of population frequencies

To compute CNV frequencies, we defined two reference sets of “founder” genomes—the parents from a large collection of trios—not known to be related. These sets included: (a) the genomes of 1584 individuals sequenced using CGI technology, and (b) the genomes of 1669 individuals sequenced using Illumina technology. These are subsets, respectively, of the ITMI-CGI and the ITMI-Illumina sets.

For each genomic segment resulting from the HMM-based segmentation, we computed the median number of individuals (in the corresponding reference set, CGI or Illumina) with the same level of coverage as observed in an individual (e.g., hemizygous), and hence the genotype frequency. We also computed the allele frequency by integrating the ploidy observations across all genomes in the reference panel.

### Comparison to the “Gold Standard” NA12878 genome

We obtained an updated assembly of the NA12878 genome from CGI, sequenced using CGI's library v.2 format and processed using version 2.5 of CGI's analytic pipeline. We analyzed this genome assembly's coverage to compute its NCP and to determine predicted deletions and CNVs by HMM segmentation. We obtained deletion calls for this genome from Supplementary Table 4 in Mills et al. (2011). These deletions were discovered and validated using a variety of methods. We translated these deletions to GRCh37 coordinates using liftOver (Hinrichs et al., 2006). Each deletion spans one or more bins, each of which may have a different normalized coverage value (i.e., the fraction of expected diploid coverage, prior to segmentation into states): we computed the median of these values as the representative normalized coverage level for each deletion.

### Evaluation of CNV calls by complete genomics

CGI's standard analysis pipeline computes predicted boundaries (junctions) of CNVs and other SVs, reported in the “highConfidenceJunctionsBeta” file. We observed that the same or very similar junction coordinates are reported in many CGI assemblies. We collected all junctions from the set of 1584 “founder” genomes and used a distance cutoff of 400 bp to cluster them into recurring junction ranges. We then computed for each such range the fraction of assemblies with a stated SV junction in that range: this serves as a metric for population frequency of the junction or propensity for false calls.

We selected events representing deletions and duplications from the file “highConfidenceJunctionsBeta” of each assembly. Since older versions of CGI's pipeline do not make this determination explicitly, we selected events with both junctions on the same chromosome, on the same strand, and within 1 Mb of each other. We annotated each deletion or duplication event with the frequency of its junctions, and computed the median NCP as above (Section Comparison to the “Gold Standard” NA12878 Genome).

### Evaluation by concordance in trios

To evaluate concordance in a family trio (father, mother, and child), we analyzed each genome in the trio independently, and then computed the total number of bins in which the child's state (ploidy) was consistent with expectation from the parents' states, as enumerated in Supplementary Table 1. Similarly, we computed total length of the segments where the offspring's state was not concordant with the parents' states. For example, if one parent is hemizygous (state 1) and the other parent has the expected diploid coverage level (state 2), expected levels for the child includes hemizygous and diploid (states 1 and 2). Any other state observed in the child would be counted as discordant. We further computed the fraction of the genome in which all family members are in state 2, and the fraction of the genome in which discordant observations within the trio involve higher copy numbers (states 3 and 4). All these computations excluded chromosomes X, Y, M and gaps in the reference genome.

Having identified discordant bins for each trio, we observed that some bins were frequently discordant in many trios, and they tended to cluster into segments. Most of these segments display also excessive heterozygosity (not shown); these represent “compressions” of the reference sequence (Roach et al., 2010), many but not all of which have been resolved in the latest version (GRCh38) of the reference sequence. We classified segments observed as discordant in 100 or more trios—totaling 3331 kb of sequence—as recurring false positive results and excluded them from further analysis.

To evaluate the specificity of the concordance metric, we created shuffled trios by selecting for each child a randomly picked father and a randomly picked mother—ensuring these are not the true father and mother for the child. We then compared each child to the replacement parents to compute the expected concordance level from trivial similarity between individuals.

### Implementation and availability

We implemented the genome coverage analysis pipeline in the Perl programming language. The code, documentation, and resources are available at http://db.systemsbiology.net/gestalt/coverage/. All the tools have very low memory requirements. Condensing the coverage signal takes a couple of hours per genome, depending on the computing speed of the machine. All other steps take a couple of minutes each.

## Results

### A modular method for coverage analysis

**Figure 1:**
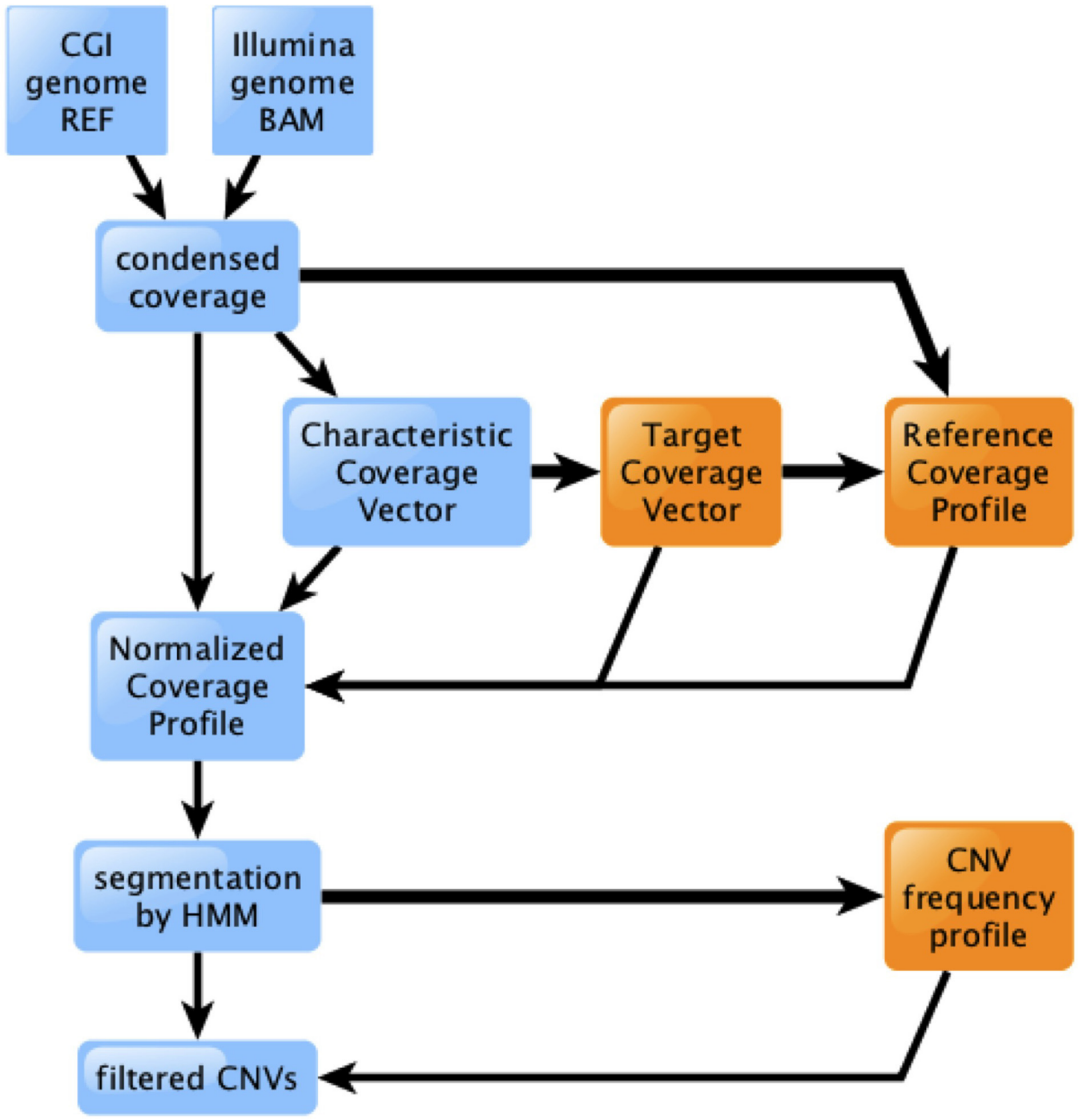
**Overview of the coverage analysis method**. Raw coverage data from CGI or Illumina technology is processed into a condensed format, then scaled, normalized, segmented, and filtered. Light blue boxes and thin lines represent data processing for a single genome. Orange boxes and thick lines represent information flow involving multiple genomes.

We have developed a new method for identification of deletions and CNVs in personal genomes, based on WGS depth of coverage. The method involves several modular stages, diagrammed in Figure 1.

**Figure 2:**
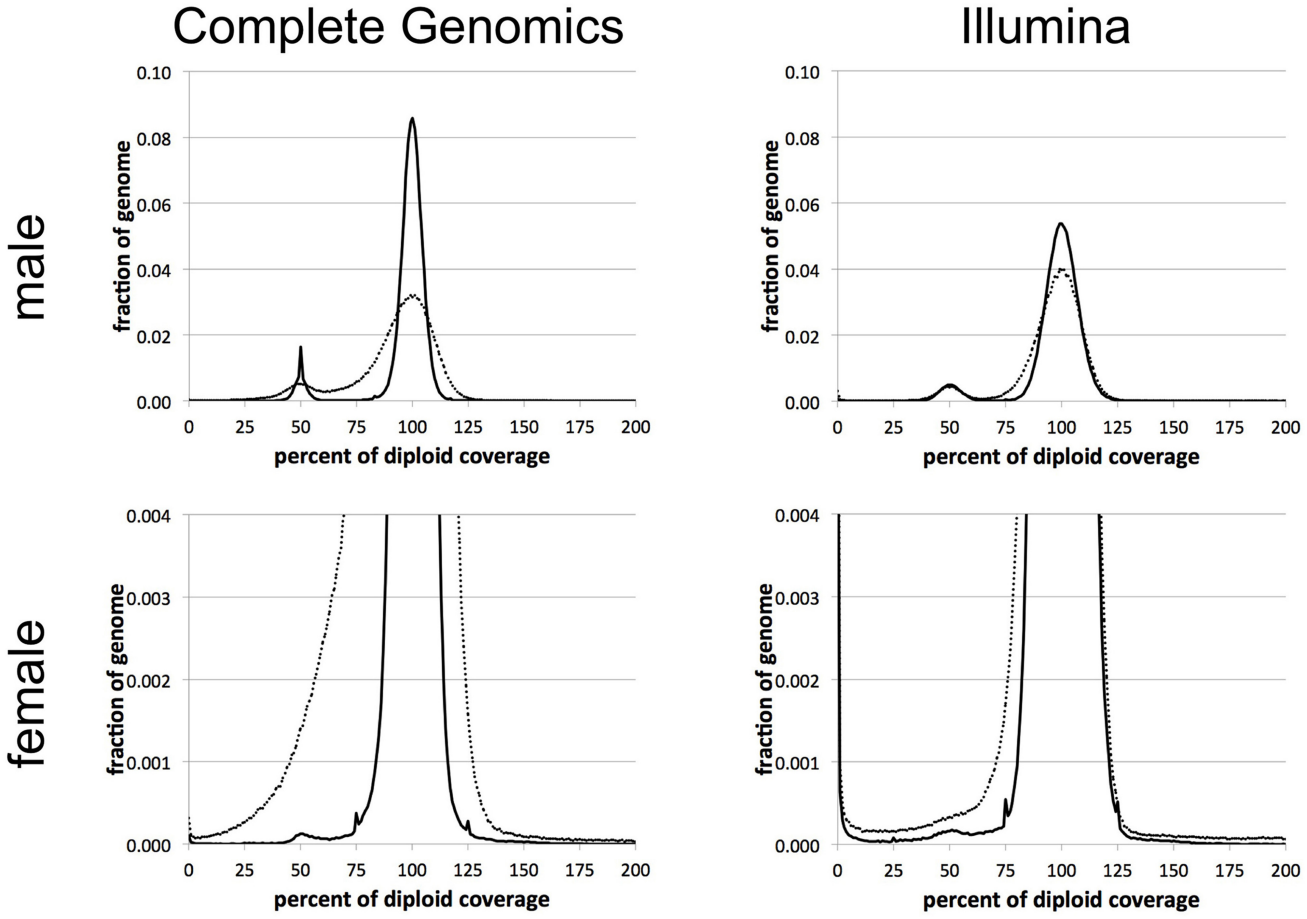
**Reference normalization sharpens individual genome coverage distributions**. Each graph represents the fraction of the genome as a function of coverage level, before normalization (dotted curves) and after normalization (solid curves). Male genomes have the expected 50% (haploid) peak representing coverage in the sex chromosomes. Female genomes have a very small peak at 50%, from (mostly autosomal) hemizygous segments. The plots for female genomes are rescaled to better visualize this peak at 50%, which is only evident after normalization.

1. We first condense the genome coverage information into an efficient, technology-agnostic format. We discuss this further in Section An Efficient Format for Storing Coverage Information.
2. We then scale the genome's coverage, partitioned by %GC, according to a pre-computed Target Coverage Vector that is characteristic of the technology and pipeline version (see Materials and Methods).
3. We normalize the scaled genome coverage to the corresponding RCP (see Materials and Methods). The resulting Normalized Coverage Profile (NCP) offers significantly improved ability to distinguish between segments of the genome that have the expected diploid level of coverage, and those that are hemizygous or nullizygous (Figure 2). This effect is more pronounced for genomes sequenced using CGI's technology than for those sequenced using Illumina; we discuss this further in Section Depth of Coverage is Consistent from Genome to Genome.
4. We finally segment the NCP using an HMM, and identify deletions and CNVs of interest by comparison to their population frequency profile (see Materials and Methods).

### An efficient format for storing coverage information

Using standard genome analysis tools, it is possible to produce detailed information on the depth of coverage at single-base resolution. CGI' standard WGS pipeline reports coverage information in a per-base long format that includes the raw coverage and (for pipeline versions 1.10 and later) the %GC-corrected coverage. This information is provided by CGI in the “REF” directory—a standard component of every delivered genome. For Illumina genomes (or any technology that produces BAM files), equivalent raw coverage information can be extracted using samtools (Li et al., 2009). Both these sources (REF and BAM) are very large (gigabytes per genome, Table 1) and thus difficult and expensive to store, transmit, and analyze. They are also frequently discarded in favor of more processed (and condensed) representations of variants relative to the reference genome. This effectively discourages detailed analysis of coverage, potentially leading to missing important discoveries.

**Table 1:** **Typical range of file sizes from which coverage information can be derived, for the CGI and Illumina technologies, as well as the sizes of “condensed” and “normalized” coverage formats, per genome**.

**File type**	**Resolution**	**Complete genomics**	**Illumina**
Raw coverage	1 bp	REF: 16.63 ± 1.56 GB	BAM: 84.42 ± 27.57 GB
Condensed coverage	20 bp	110.37 ± 3.72 MB	89.58 ± 2.53 MB
NCP	1 kb	9.29 ± 0.18 MB	9.38 ± 0.06 MB
Variants		var: 279.48 ± 26.26 MB	gVCF: 2.51 ± 0.71 GB

*The sizes of files representing variants are included for comparison. NCP, normalized coverage profile*.

**Figure 3:**
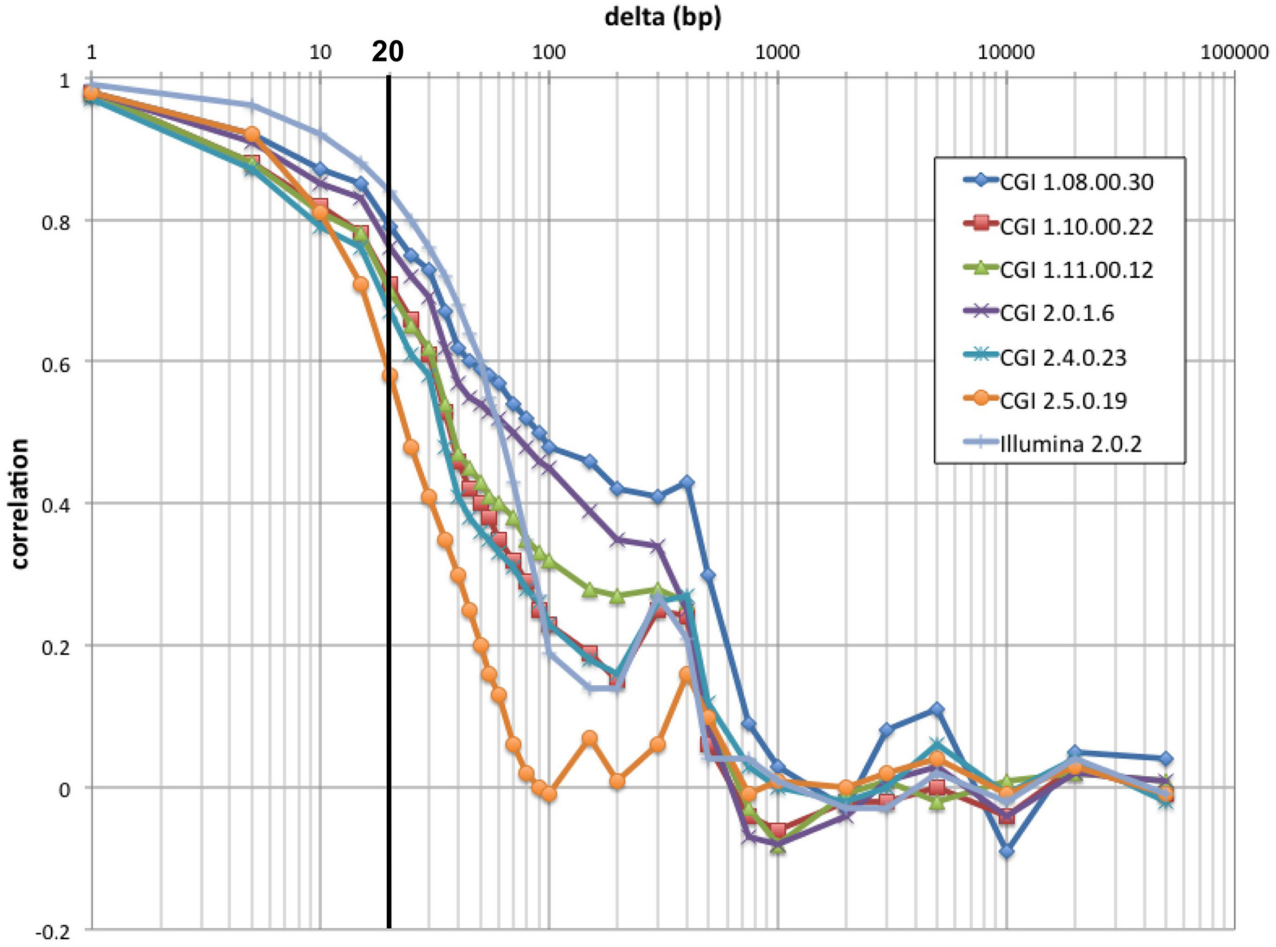
**Raw coverage autocorrelation**. For a 100 kb segment of typical sequence composition (chr1:9,010,001–9,110,000), autocorrelation along the genome of raw coverage (prior to scaling) is shown as a function of distance for seven individual genomes: six from distinct CGI pipeline versions and an Illumina genome. As expected, short-range autocorrelation is higher for the technology with longer reads (Illumina). Past the read length, autocorrelation decreases sharply; except for older CGI pipeline versions, it rises again approaching insert (300–400 bp). At larger distances (>750 bp), coverage is essentially uncorrelated. The bold line indicates the compression cutoff chosen (20 bp).

We have devised a compact representation of the coverage trace of a genome. Since each sequence read spans several consecutive positions along the genome (typically 30–35 for CGI, 100–250 for Illumina, from 300–400 bp inserts), we reasoned that the coverage signal should show significant short-range correlation and thus may be compressed with little loss of information. Autocorrelation analysis (Figure 3) confirmed that depth of coverage is autocorrelated at least 50% over half a read length, with additional but lower correlation consistent with the separation between insert ends. We chose to bin coverage in 20 bp windows; at this distance, autocorrelation ranges from 0.58 to 0.84 depending on technology and pipeline version. The correlation is even higher between positions located within a single bin. We further compressed the signal by using progressively lower resolution for high-coverage values (see Materials and Methods). This encoding method reduces the representation of coverage by ~150-fold for CGI genomes, and to 0.1% the size of a typical BAM file for Illumina genomes (Table 1).

### Depth of coverage is consistent from genome to genome

Depth of coverage across the genome strongly depends on the sequencing technology used, and to a lesser extent, on the version of the technology. We therefore stratified our training genome assemblies into 10 chronological groups of CGI pipeline versions, from the earliest released to the most current (Supplementary Figure 1), and analyzed assemblies for each group as well as assemblies sequenced using the Illumina platform separately.

We estimated the (technology- and version-specific) diploid level of coverage at each position (1 kb bin) in the genome from the observed distribution of scaled coverage in individuals. This metric is equivalent to the median coverage value for most genomic bins, and is robust to the presence of outliers. The estimated diploid level deviates from the median in the presence of common deletions and CNVs in the population; below, either term refers to the estimated diploid level.

**Figure 4:**
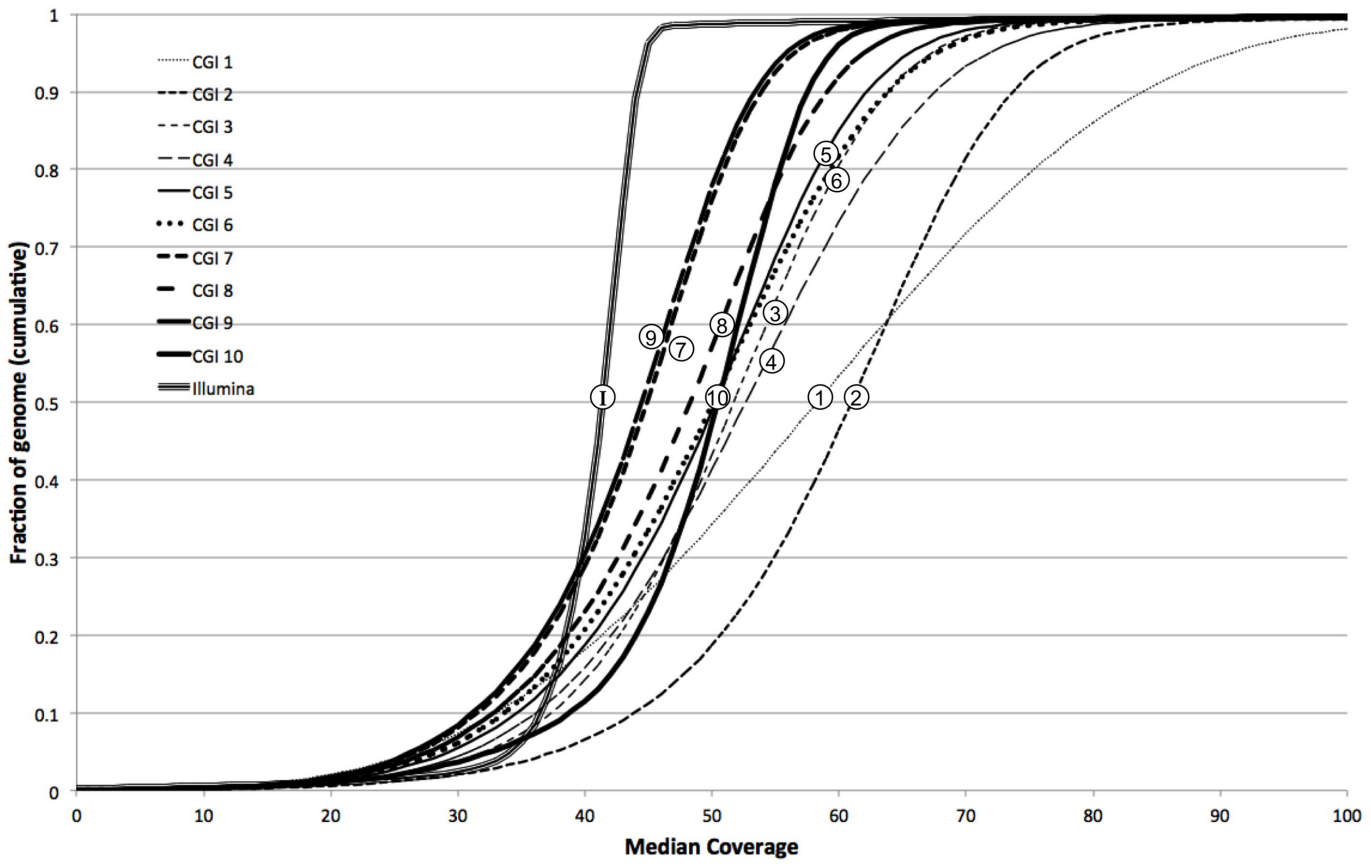
**Illumina genomes are more uniform in coverage**. Cumulative fraction of genome as a function of median coverage for 10 CGI pipeline versions and Illumina: more uniform coverage across a sample results in sharper, step-like sigmoidal curves.

**Figure 5:**
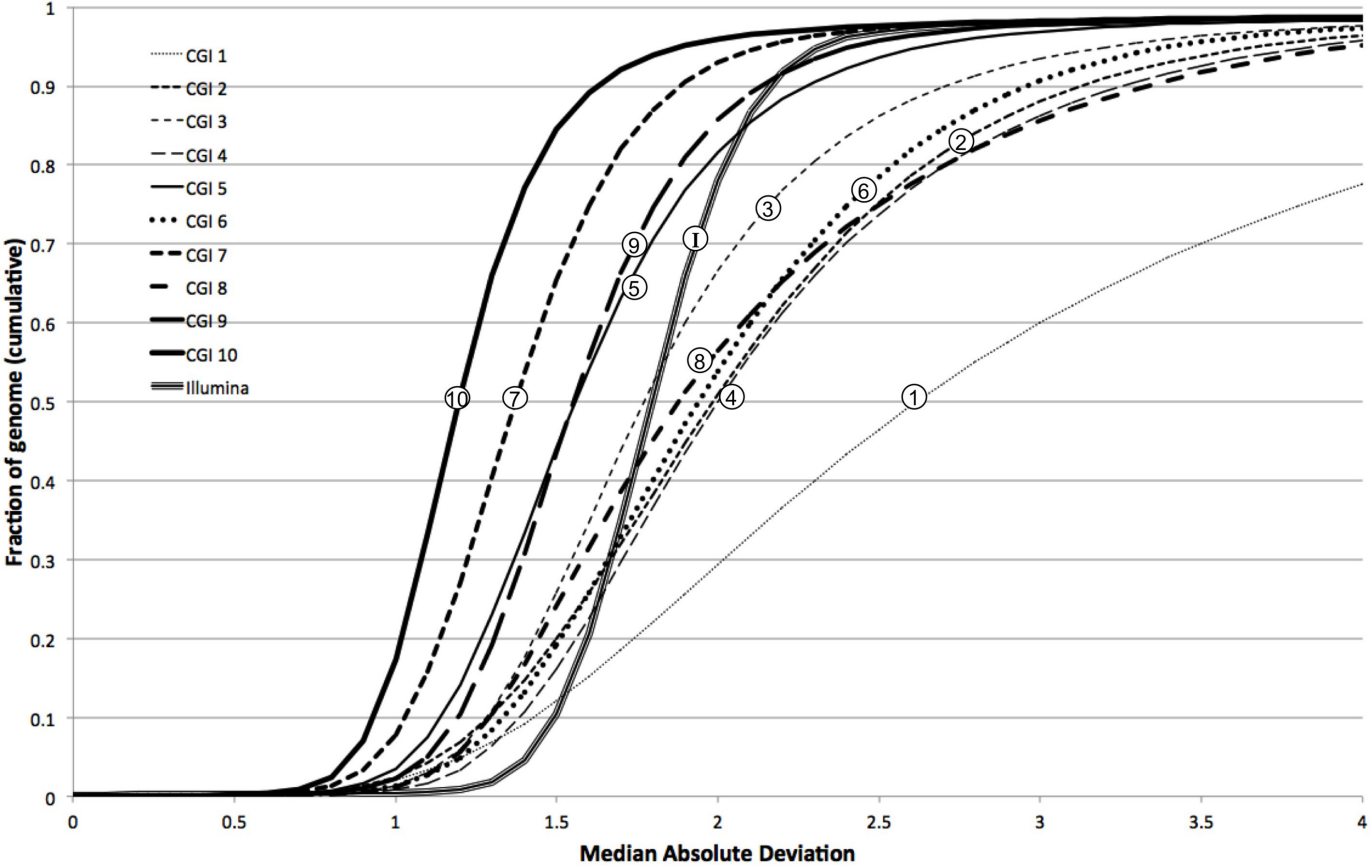
**Modern CGI genomes are more consistent from sample to sample**. Cumulative fraction of genome as a function of median absolute deviation for 10 CGI pipeline versions and Illumina: curves closer to the left have more consistent coverage among samples.

We further characterized the variation in coverage among genomes, at each position, using the median absolute deviation (MAD) from the median coverage. Based on the genome-wide distribution of these two metrics (estimated diploid level and MAD), we assessed the uniformity of coverage within and among genomes. We observed that earlier versions of the CGI technology had very large variation in coverage levels within genomes, though this variation has sharply decreased in more modern versions (Figure 4). We observed much higher uniformity of median coverage *within* genomes sequenced on the Illumina platform. On the other hand, we observed much more consistent coverage *among* genomes sequenced with CGI's current technology than among Illumina genomes (Figure 5), even though the latter were sequenced using the same version of the technology and processed using the same pipeline versions. The consistency between Illumina genomes sequenced using different read lengths, on different machines, and processed with other software tools remains to be determined. We again observed a general trend of improvement (reduced technical variation from genome to genome) from the older to the newer versions of CGI's technology.

In other words, whereas the depth of coverage fluctuates much more strongly along a single CGI genome assembly than along a single Illumina genome assembly, the fluctuation is much more consistent and predictable from one CGI assembly to another than from one Illumina assembly to another. These results suggest that computational methods for detection of CNVs not explicitly correcting for locus-specific coverage differences (i.e., based on the expectation that coverage follows a common distribution genome-wide) should be more useful for analyzing genomes sequenced on the Illumina platform than when interpreting CGI genomes. Conversely, the very consistent coverage observed among CGI genomes suggests an opportunity for improving CNV detection by normalizing each genome's coverage to a pre-computed profile of empirically derived reference values—the method we present here.

**Figure 6:**
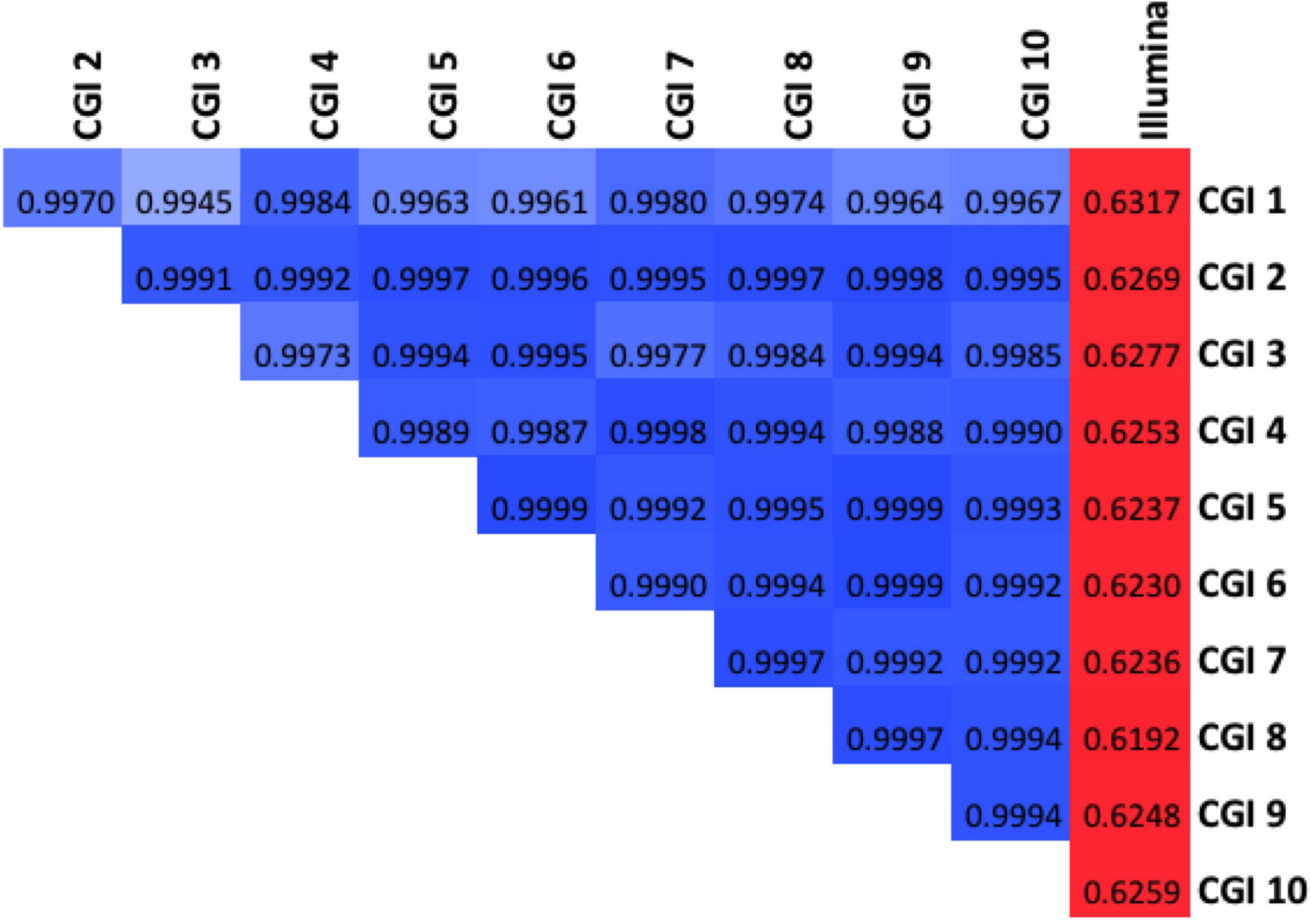
**The Reference Coverage Profiles are very highly correlated**. Global correlation among the 11 RCPs evaluated (10 CGI, one Illumina). Darker blue shades denote higher correlation above 0.99; red shades represent correlations in the 0.5–0.9 range.

We find the RCPs computed from each of these 11 groups of assemblies are all highly correlated (Figure 6). As expected, the correlation between the Illumina RCP and any CGI RCP is much lower (*r* ~ 0.63) than between any pair of CGI RCPs (*r* > 0.99). Likewise, the very earliest CGI pipeline versions yield RCPs that are slightly less similar to the more modern CGI versions.

### Concordance within trios

We evaluated the performance of the coverage normalization method by quantifying NCP concordance within 836 family trios (father, mother, and child) sequenced using CGI's technology. For each trio, we identified segments in which the called coverage level (HMM state) in the child is consistent with the corresponding calls in the parents. Segments with unexpected combinations of states represent either *de novo* CNV changes (expected to be rare), errors in the reference sequence (which we excluded, see Materials and Methods), normalization errors or, if observed frequently throughout the genome, incorrect family relationships.

The observed concordance across all trios was very high, spanning 99.93% ± 0.024% of the genome. The lowest observed concordance in a trio was 99.83%. Since most of the genome is diploid in most people, this metric is also high when comparing the child to randomly picked parents (99.79% ± 0.274%).

A much stricter metric of concordance excludes from the computation all regions in which father, mother, and child are in state 2 (normal diploid coverage). Using this metric, the observed concordance across all trios was 87.75% ± 5.04%. With randomly picked parents, strict concordance was reduced to 66.27% ± 8.73%.

### Evaluation of deletions in the NA12878 genome

The NA12878 genome has been extensively analyzed and serves as a “gold standard” genome for technology and algorithm development. We analyzed a recent assembly of NA12878, sequenced with CGI's current technology, and compared the resulting NCP with results of previously published analyses (Mills et al., 2011; Layer et al., 2014).

**Figure 7:**
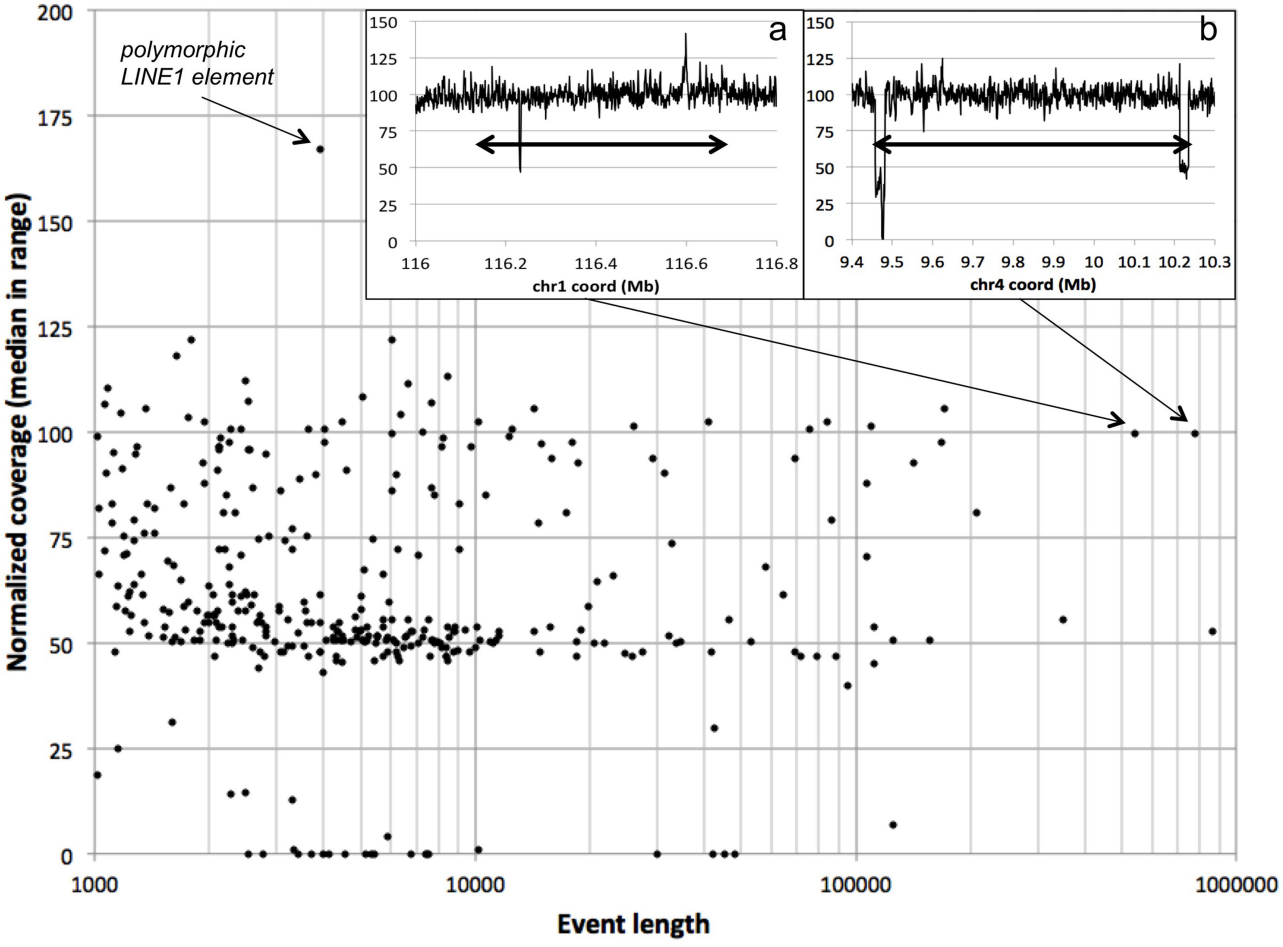
**Deletions in the NA12878 genome**. For each “validated” deletion in the NA12878 genome, we evaluated the median normalized coverage (100 represents diploid level, 50 represents hemizygosity, etc.) vs. the length of the event. A polymorphic LINE1 element stands out as a high-coverage outlier. Insets: normalized coverage traces for the two longest “validated” deletions showing near diploid level median coverage; double-headed arrows denote the spans of the reported deletion events.

We use 75% normalized coverage as the least stringent (highest) cutoff for separating deletions (hemizygous and nullizygous) from the “bulk” diploid coverage (Figure 2). We assessed the NCPs over each of 361 previously-reported autosomal deletions in NA12878 by computing the median NCP within the reported range. We found that 262 of these (73%) have median normalized coverages lower than 75% (Figure 7). As expected, we observe more variability of median NCP for the shorter deletion calls, due to the 1-kb bin size used in our study. One outlier 4-kb deletion call with excessive coverage corresponded to a polymorphic LINE1 element, hinting at read-mapping errors. We evaluated the longer validated deletion calls with ~100% normalized coverage by our method as potential false negatives. We found that the longest such deletion (chr4:9,461,230–10,235,268 in GRCh37 coordinates, 774 kb) is flanked by two segments of reduced normalized coverage (24 kb and 22 kb long) consistent with hemizygosity (inset b in Figure 7). We hypothesize that this may have resulted in a deletion miscall of the entire 774 kb span by other methods. The second longest potential false negative (chr1:116,135,317–116,677,627 in GRCh37 coordinates) shows quite consistent normalized coverage throughout its 542 kb span (inset a in Figure 7). Neither of these two large deletions was identified by LUMPY (Layer et al., 2014), suggesting that these deletions were false positives in the published set rather than false negatives for our method.

We similarly evaluated a much richer set of CNV events identified by LUMPY (Layer et al., 2014) on the NA12878 genome. LUMPY's integrative method allows detection of very short CNVs, shorter than our current analytical resolution; we therefore evaluated only 736 CNV calls at least 1 kb long (Supplementary Figure 6). Of these, 53 have lengths of 6.0–6.3 kb, consistent with full-length LINE1 elements. We similarly observed a large number of reported CNVs ~300 bp long (under our 1 kb cutoff, and thus not shown), consistent with full-length *Alu* repeats. Both LINE1 and *Alu* repeats commonly lead to false positive findings due to the presence of very large numbers of them in the genome, and to mismapping of reads derived from them. We found that 430 LUMPY CNV calls (58%) have median normalized coverage lower than 75%. This fraction rises to 75% (118 of 157) when considering events longer than 5 kb and excluding LINE1-sized events.

### Concordance with CGI's CNV calls

**Figure 8:**
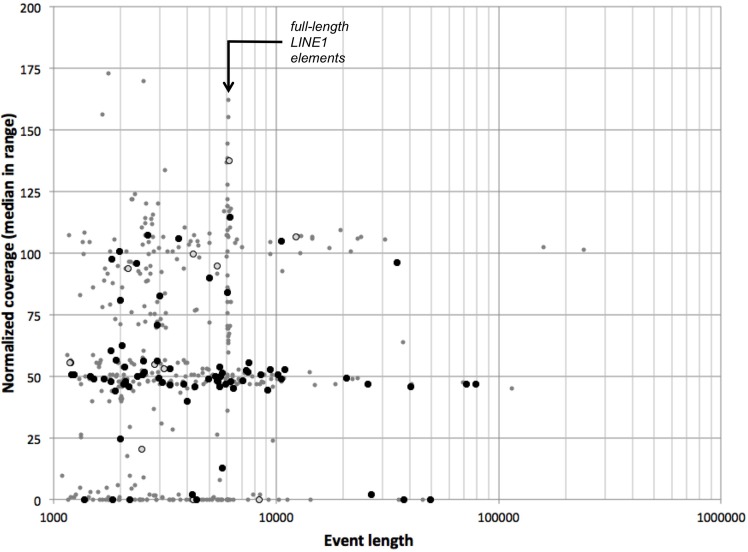
**CGI CNVs in the NA12878 genome**. For each deletion or duplication event in the NA12878 genome called by CGI, we evaluated the median normalized coverage (100 represents diploid level, 50 represents hemizygosity, etc.) vs. the length of the event. Black circles: events with boundaries outside recurring junction clusters. Open circles: events with one boundary outside recurring junction clusters. Gray points: all other events.

Complete Genomics' analysis pipeline includes a detailed analysis of SVs, including deletions, inversions, tandem, and distal duplications, as well as complex and interchromosomal events. We evaluated population frequency and representative coverage of 470 events representing deletions and duplications over 1 kb long in the NA12878 genome (see Materials and Methods); 51 of these have lengths of 6.0–6.3 kb, consistent with full-length LINE1 elements (Figure 8). We found that 328 CNVs (70%) have median normalized coverages lower than 75%—as expected for hemizygous or nullizygous deletions. Requiring that both junctions of a CNV be infrequent in the population (frequency less than 0.1 each) enriches this proportion to 84% (63 of 75 events); only one LINE1-sized deletion passes these filters. We further verified that hemizygous deletions are flanked by segments of expected diploid coverage, and nullizygous deletions by diploid or haploid coverage (not shown).

### Distribution and effects of rare deletion events in genomes

We studied the distribution of rare (frequency < 0.01) hemizygous and nullizygous deletions at least 3 kb long, in the autosomes of 1584 unrelated genomes sequenced using CGI technology and 1669 unrelated Illumina genomes. We observed that, on average, each CGI genome presented 11.2 segments in hemizygous state and 2.2 segments in nullizygous state. Illumina genomes had very similar hemizygous deletion frequency (10.9 segments/genome) and somewhat higher nullizygous frequency (3.6 segments/genome).

We further assessed how frequently genes are affected by these rare deletions. We defined as “affected” a gene in which at least one annotated exon of at least one transcript is fully or partially contained in a deleted segment. Not all exons are constitutive (included in all transcripts of a gene). Considering the diversity of alternative splicing forms, which may be differentially expressed in various tissues and cell types, not all exon deletions need result in an observable phenotype. Since we use a 1-kb bin size, we excluded the terminal bins of each deletion from this computation, to maximize the probability that the exon is indeed disrupted by the deletion event. When analyzing the CGI “founder” individuals, we found that 1437 autosomal genes (distinct genes in the UCSC Genome Database, track kgXref) contain a fully or partially hemizygous exon in at least one individual, and 84 are “knocked out” (at least one nullizygous exon) in at least one individual (Supplementary Figure 7). Similarly, Illumina “founder” individuals presented 1404 autosomal genes with at least one hemizygous exon, and 189 genes with at least one nullizygous exon.

Conversely, we found that most individuals sampled (73% CGI, 74% Illumina) harbored at least one gene with an exon in hemizygous state from a rare deletion. As expected, much fewer (11% CGI, 28% Illumina) had at least one gene with an exon “knocked out” (Supplementary Figure 8).

## Discussion

Many approaches for CNV discovery from second generation short-read re-sequencing data have been explored. Nevertheless, such analyses are not routinely performed on personal genome data, for a variety of reasons. Most methods for CNV discovery have been designed to work on Illumina data (or equivalent); working with CGI raw data is much more difficult due to the fragmented structure of the sequence reads. In all cases, the data files required for analysis are very large—tens to hundreds of gigabytes in size.

We have developed a method for compressing the coverage information in personal genomes down to a very manageable file size, which should pose no more difficulty for storing and transmitting over networks than the standard files used to describe sequence variants. We encourage researchers to apply this conversion to their genome data to facilitate downstream analyses.

The depth of coverage fluctuates strongly from locus to locus, affected by %GC, mapability, and other sequence-specific patterns, which may be technology-specific. Coverage may also change from locus to locus in actively replicating cells (e.g., cell lines and cancer samples): for this reason, we restricted our analyses to DNA derived from blood and saliva (buccal cells). Even lacking an *ab initio* model of all these effects, the depth of coverage along the genome can be empirically modeled by comparing coverage profiles among samples, leading to significantly improved CNV calls (Klambauer et al., 2012). This again poses a technical challenge, compounding the difficulty managing and analyzing coverage information from individual genomes. Furthermore, suitable “control” genomes may not be available, particularly in a clinical context.

We presented here a solution to this difficulty, by way of pre-computed multi-genome RCPs. Comparing one genome to a pre-computed reference is conceptually equivalent to analyzing a set of hundreds or thousands of genomes simultaneously—but while the former is technically easy, the latter is essentially intractable for coverage analyses. We stress the value of a large cohort of high-quality (>40×) genomes for training such multi-genome profiles, and the added value of the family structure of the cohort, for internal validation of parameters and results.

Depth of coverage methods can precisely quantify copy numbers in complex genomic regions (Nguyen et al., 2014), but cannot determine the actual structure of segmental duplications, nor detect balanced rearrangements. A disadvantage of CNV discovery based on depth of coverage is the lower resolution that can be feasibly achieved for detecting the boundaries of each event. While we presented here normalization and segmentation at 1-kb resolution, it is possible to increase the resolution to 20 bp—the bin size we use for condensing the coverage signal. The resources required are expected to increase linearly with the resolution. Ultimately, precise breakpoint identification requires analysis of sequencing read data.

There are many possible algorithms that could be used for computing CNVs in a genome from its Normalized Coverage profile (NCP). We presented here segmentation of the NCP using HMMSeg (Day et al., 2007). We have applied this method to thousands of genomes from a variety of cohorts, including families with individuals affected with a variety of diseases. We have identified deletions affecting genes as candidate causal mutations. For example, a 85-kb *de novo* deletion spanning the 5′ region of *NOTCH1* and causing Adams-Oliver syndrome (Stittrich et al., 2014).

The method we developed is modular by design. Its components can be used for other purposes and integrated into other pipelines. For example, the RCPs could be of use for interpreting “sequencing drop-out” regions in other uses of the short-read sequencing technologies, e.g., RNA-seq, ChIP-seq, etc. The NCP for a genome could be integrated into probabilistic frameworks such as LUMPY (Layer et al., 2014) or could be added as a track for visualization in the UCSC browser (Hinrichs et al., 2006). The CNV frequency profiles may be of use for downstream population analyses and to filter common variants, expediting the identification of causal variation in disease studies.

### Conflict of interest statement

The authors declare that the research was conducted in the absence of any commercial or financial relationships that could be construed as a potential conflict of interest.

## Abbreviations

%GC: the G+C content
CGI: Complete Genomics, Inc.
CNV: copy-number variant/variation
GRCh37: GRCh38, Genome Reference Consortium, human reference 37/38
HMM: hidden Markov model
ISB: Institute for Systems Biology
ITMI: Inova Translational Medicine Institute
MAD: median absolute deviation
NCP: normalized coverage profile
RCP: Reference Coverage Profile
SV: structural variant/variation
WGS: whole genome sequence.
