# Supplementary material for: Identification of copy number variants in whole-genome data using Reference Coverage Profiles
Source: Front Genet. 2015 Feb 17;6:45. doi: 10.3389/fgene.2015.00045 (PMC4330915; doi:10.3389/fgene.2015.00045)
Supplement: Supplementary file 1 [file DataSheet1.DOCX]

**Supplementary materials for “Identification of copy number variants in whole-genome data using Reference Coverage Profiles”, Glusman *et al.*, Frontiers in Genetics Research Topic “Marching towards 100% whole genome sequencing”**

**Supplementary Figure 1. Overview of the data set.** Distribution of assemblies studied in the ten groups of Complete Genomics (CGI) pipeline versions and the Illumina data set. Inset: proportions of assemblies derived from the Institute for Systems Biology (ISB), the Inova Translational Medicine Institute (ITMI) and CGI public data.


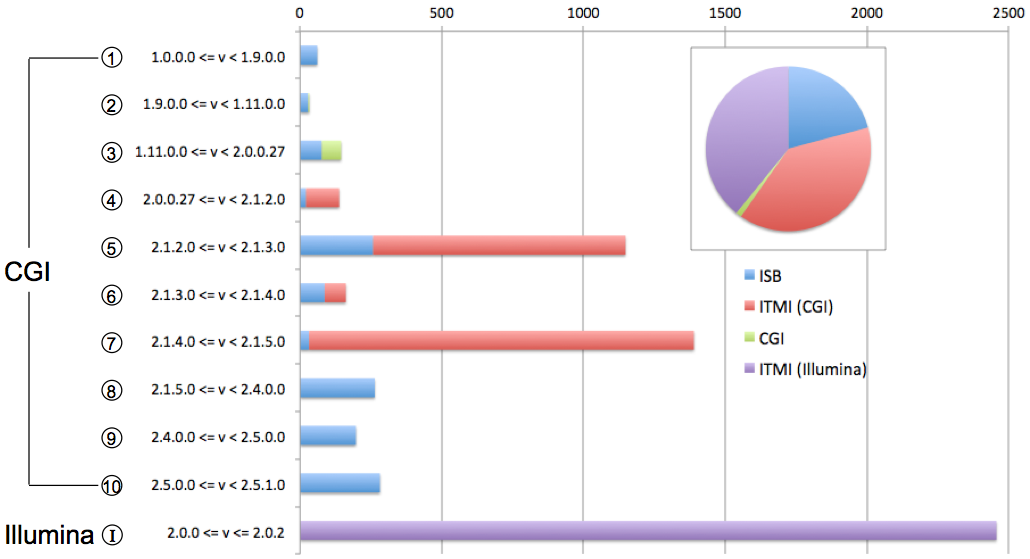


**Supplementary Figure 2. The compressed encoding system.** Values are encoded without change up to the switch value (200), then progressively lose resolution following a square root function. Inset: comparison between the original value before encoding, and the resulting value after decoding.


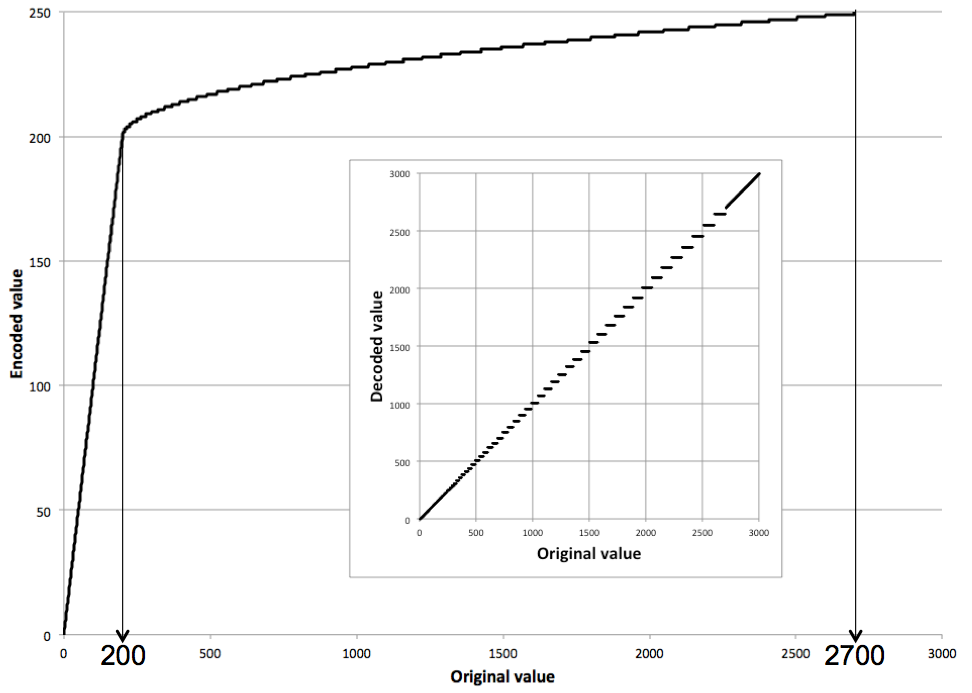


**Supplementary Figure 3. Estimation of diploid coverage level.** Shown is a complex example with assemblies at nullizygous, hemizygous, diploid and higher levels of coverage. The diploid level is estimated to best account for the observed distribution, preserving Hardy-Weinberg Equilibrium (HWE) constraints. For example, choosing 20x as diploid level would imply absence of hemizygous genomes, while observing many nullizygous and diploid genomes – in contradiction of HWE expectation.


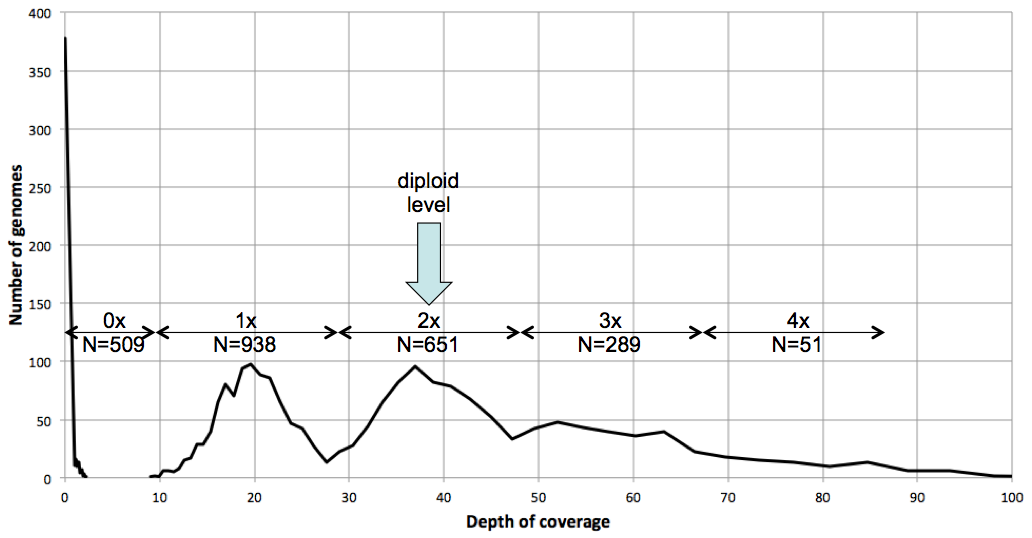


**Supplementary Figure 4. Reference Coverage vs. mapability, stratified by %GC.** We evaluated whether the estimated diploid coverage level, which we use as reference for normalization, can be predicted from a combination of %GC and mapability. We observed a very complex relationship between these parameters, leading to inability to predict the coverage from the %GC and the mapability.

We stratified the genome into 25 equal-sized %GC buckets (see Methods). We computed for each 1-kb bin the average mapability from the wgEncodeCrgMapabilityAlign100mer track, downloaded from the UCSC database, using the command:

bigWigSummary wgEncodeCrgMapabilityAlign100mer.bw –type=mean

Low mapability values are rare: 80% of the genome has maximal mapability (panel a). We plotted the estimated diploid coverage (Ref Coverage) vs. the mapability, stratified by GC bucket, for old, intermediate and new versions of CGI technology (b, c and d, respectively) and for Illumina technology (e).

We observed a complex relationship between the estimated diploid coverage and mapability, stratified by %GC:

1) For the newest CGI versions, low mapability may lead to lower diploid coverage for intermediate or very high %GC (diagonal point clouds in GC buckets 8-13 and 24), but there is a separate component seemingly unaffected by low mapability (horizontal point clouds in all GC buckets). The relation between coverage and mapability is much more diffuse for intermediate and old versions of CGI. For Illumina, lower mapability leads to a much smaller decrease in coverage, and only at the most extremely low values of mapability.

2) For high mapability levels (most of the genome), the distribution of observed coverage levels is very wide (e.g., 20-60x).


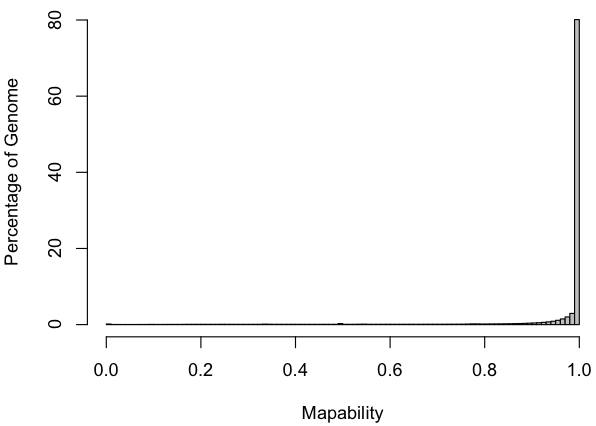


Supplementary Figure 4a: Density distribution of mapability in the genome: low mapability regions are rare.


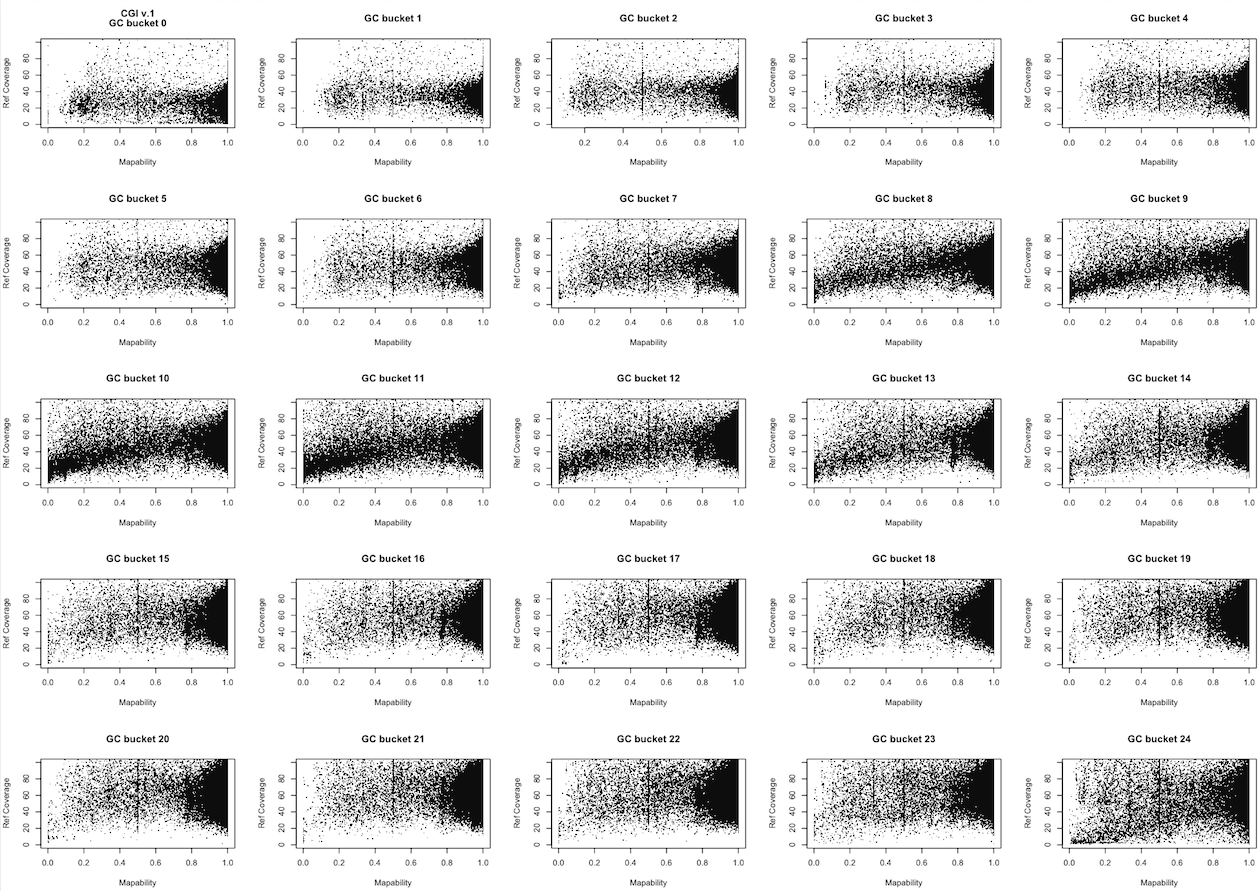


Supplementary Figure 4b: Reference Coverage vs. mapability for the oldest versions of Complete Genomics’ technology (CGI-1).


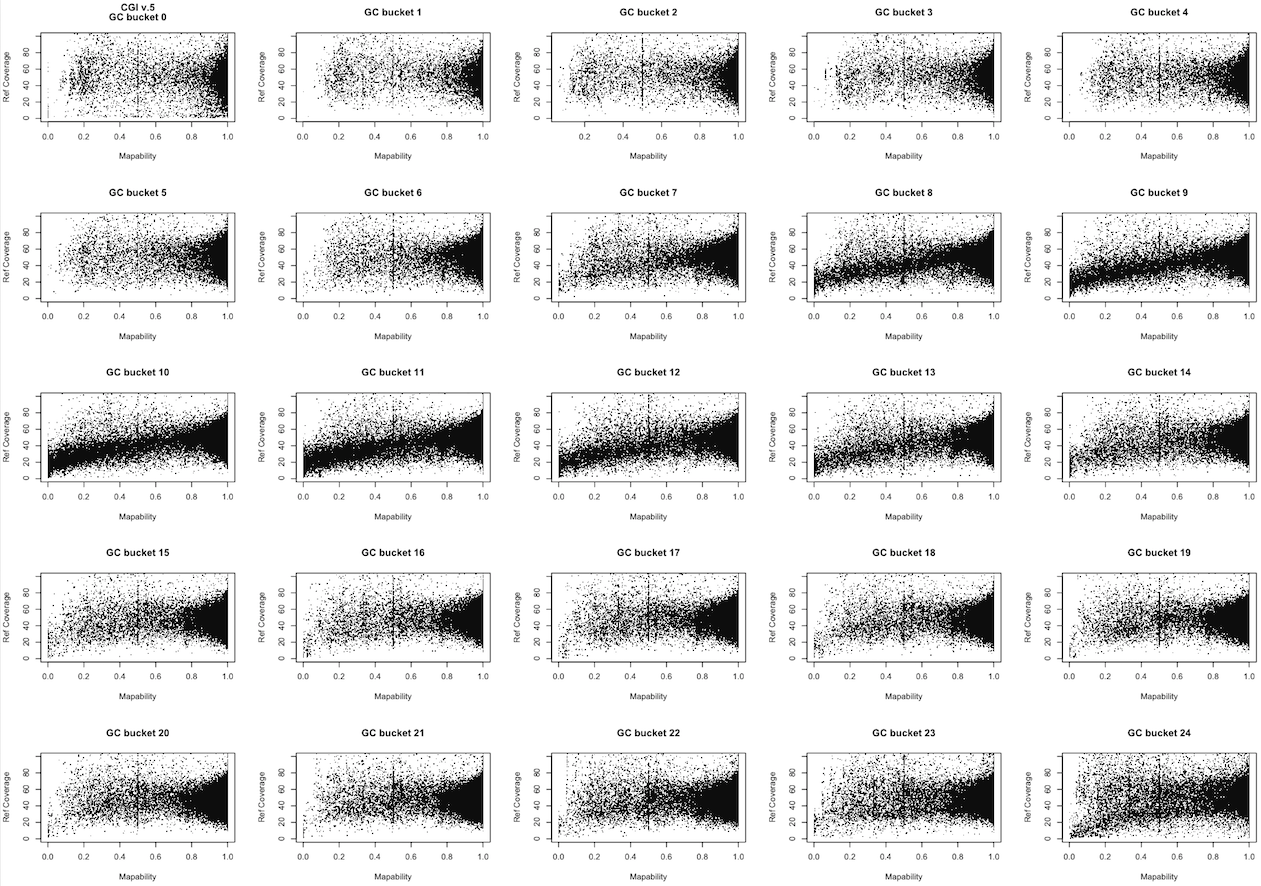


Supplementary Figure 4c: Reference Coverage vs. mapability for intermediate versions of Complete Genomics’ technology (CGI-5).


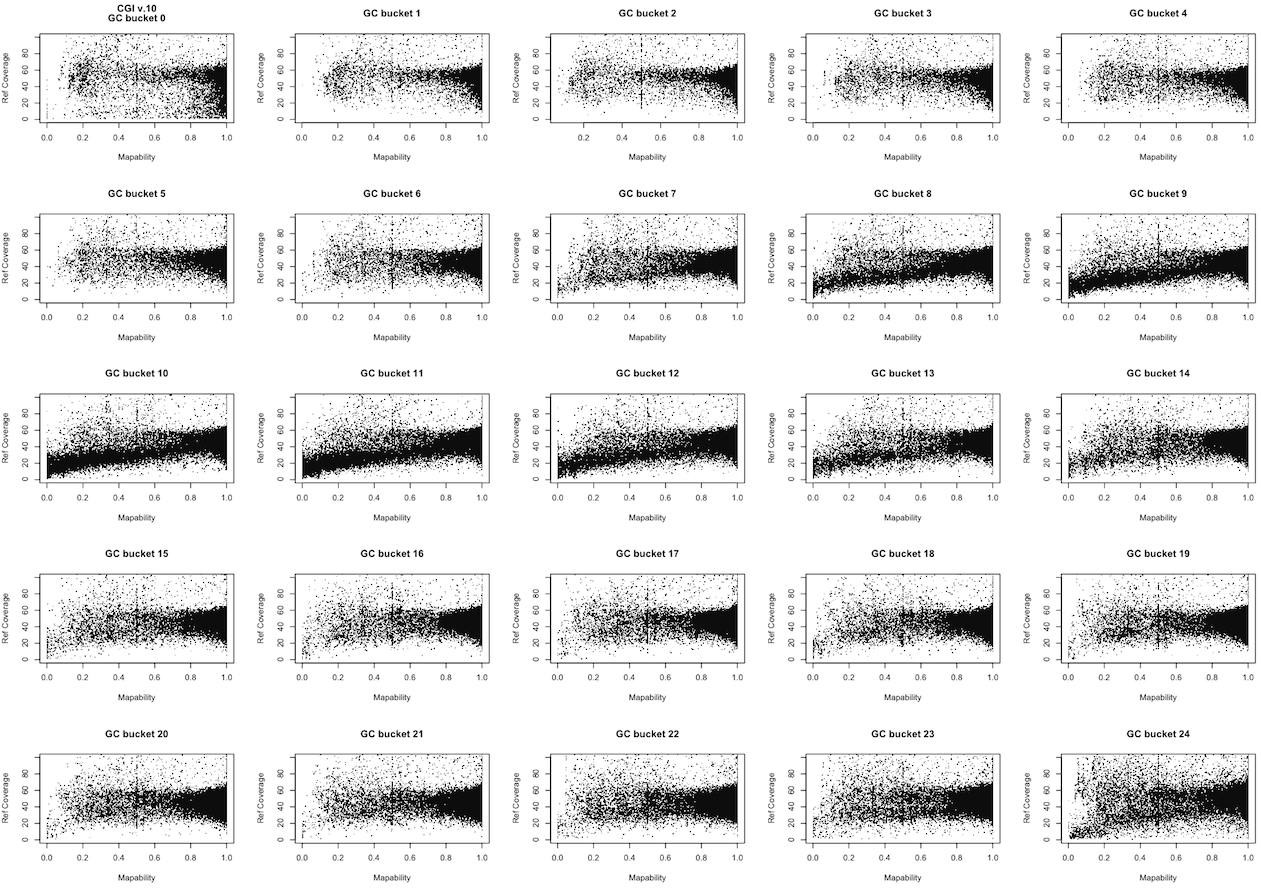


Supplementary Figure 4d: Reference Coverage vs. mapability for the newest versions of Complete Genomics’ technology (CGI-10).


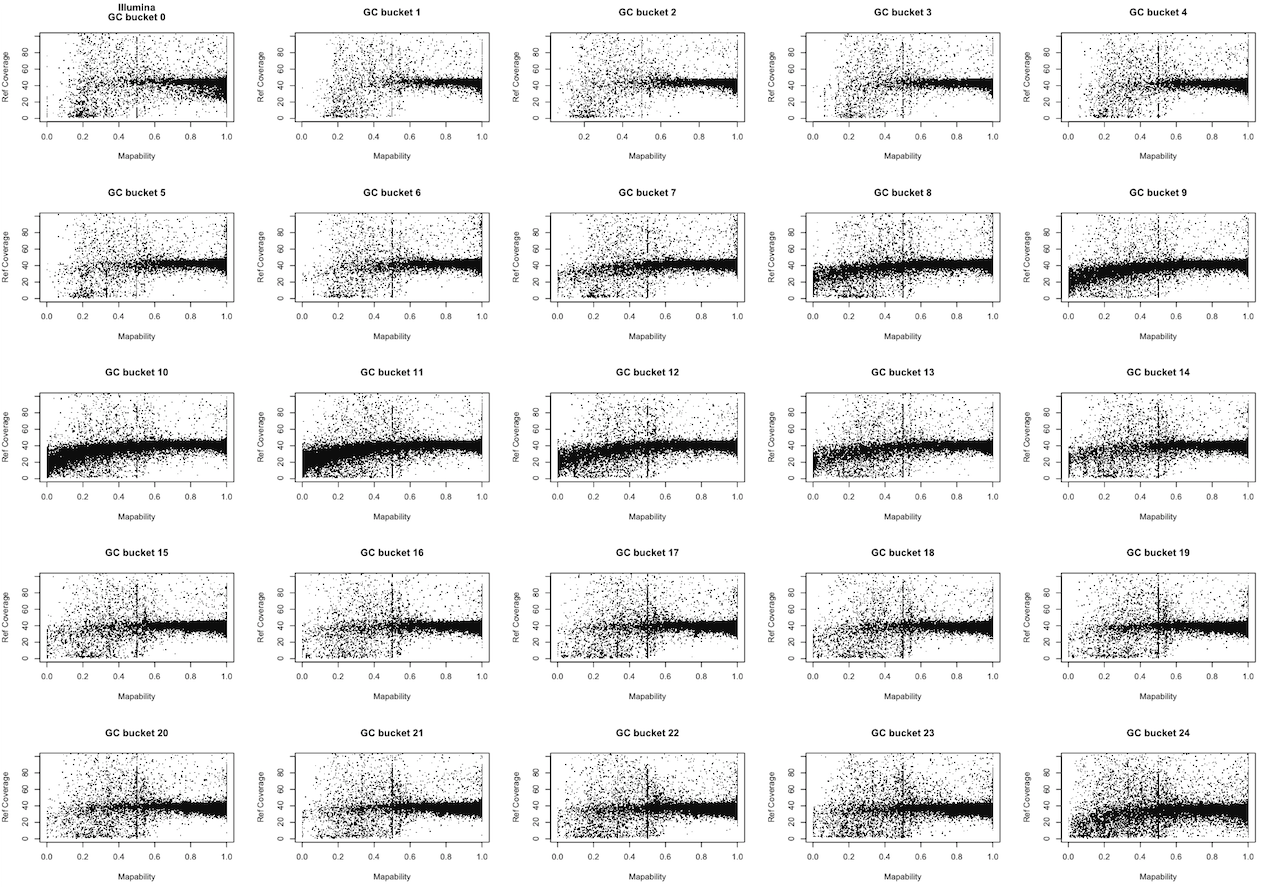


Supplementary Figure 4e: Reference Coverage vs. mapability for Illumina technology.

**Supplementary Figure 5. A five-state Hidden Markov model.** Each state has a specified Mean emission corresponding to the % of the estimated diploid level and a Variance (larger for state 2 due to its genomic prevalence, and for state 4 since it captures higher coverage levels). Arrows represent state transitions; numbers at the beginning of arrows represent transition probabilities. Dashed arrows = rare transitions. Thick arrows = most probable transitions.


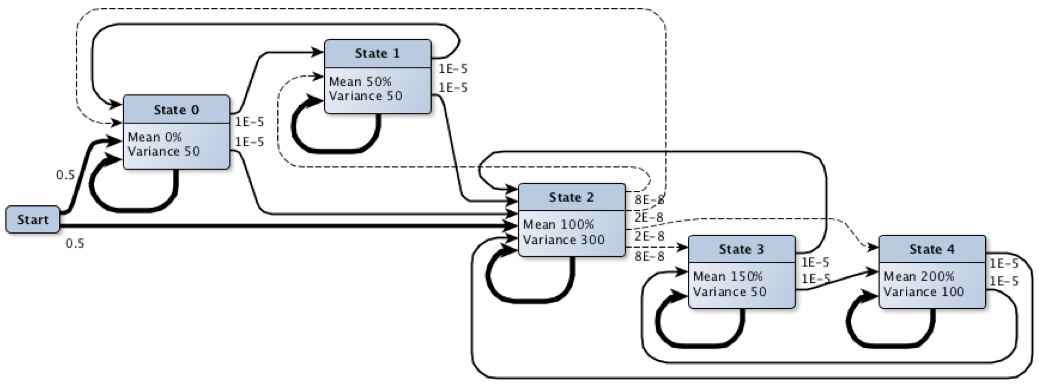


**Supplementary Figure 6. CNVs in the NA12878 genome as called using LUMPY.** For each CNV, we evaluated the median normalized coverage (100 represents diploid level, 50 represents hemizygosity, etc.) vs. the length of the event.


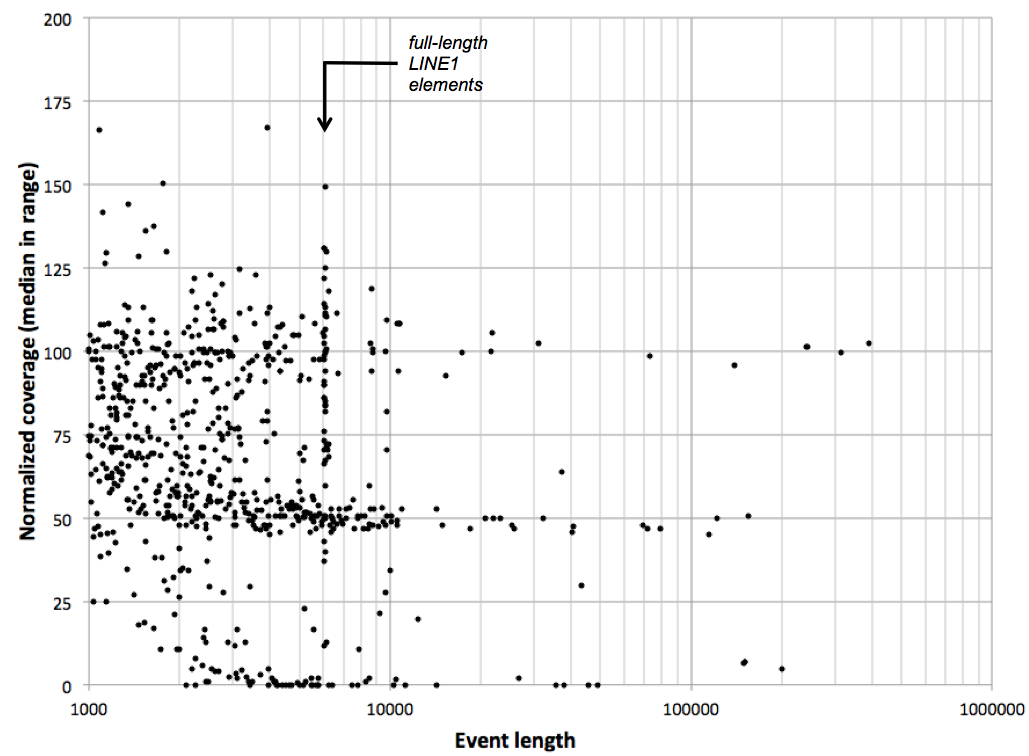


**Supplementary Figure 7. Deletions affecting genes.** Cumulative distribution of number of genes affected by deletions as a function of the number of individuals carrying such deletions. Open and filled bars represent hemizygous and nullizygous deletions, respectively. (a) CGI genomes. (b) Illumina genomes.

**
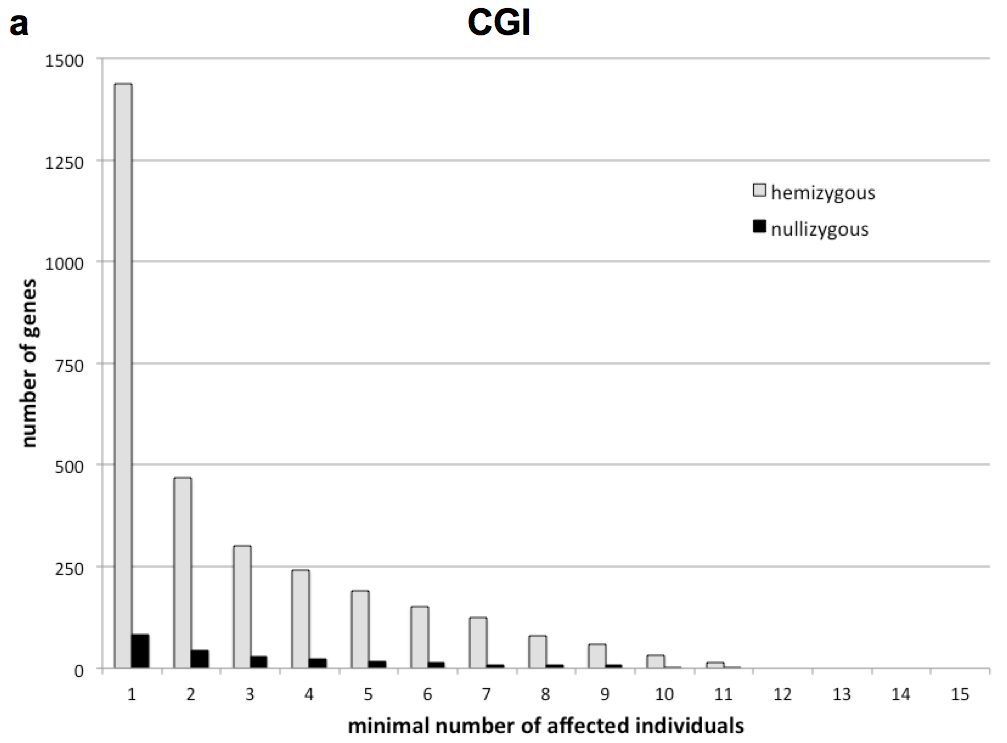
**


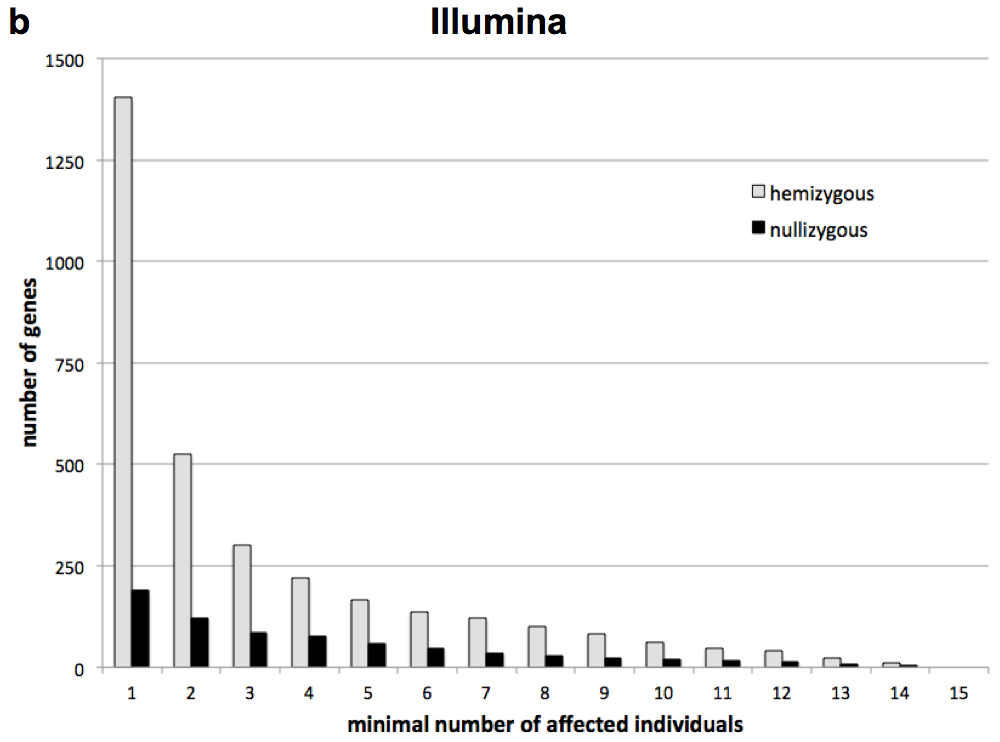


**Supplementary Figure 8. Deletions affecting genes – per individual.** Cumulative distribution of number of individuals carrying deletions affecting genes, as a function of the number of genes thus affected. Open and filled bars represent hemizygous and nullizygous deletions, respectively. (a) CGI genomes. (b) Illumina genomes.

**
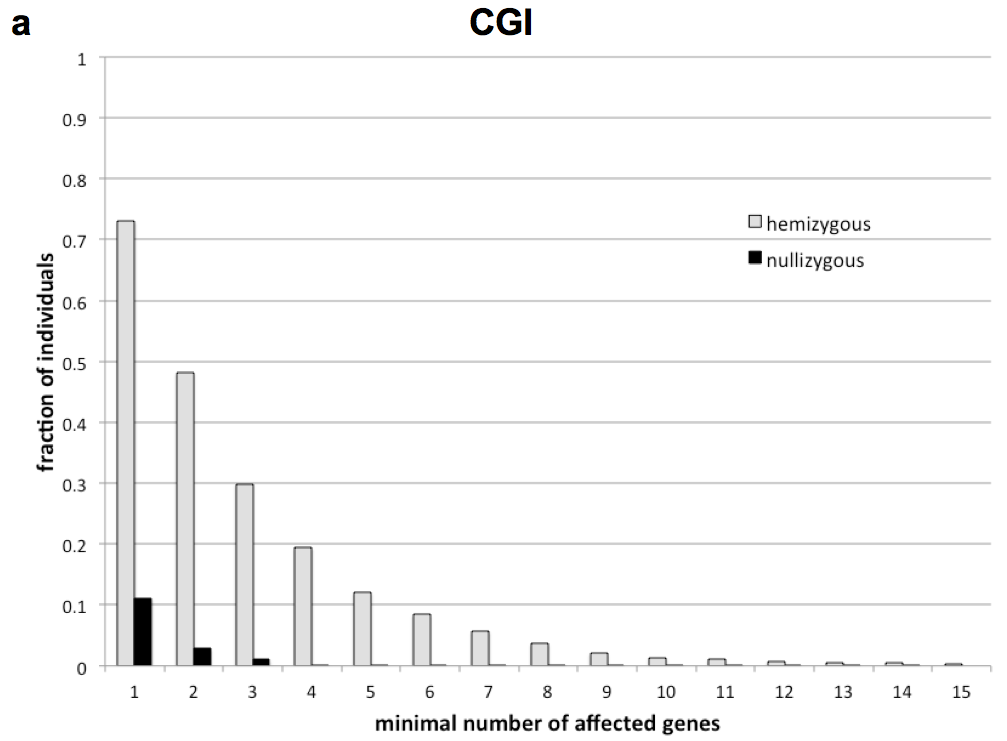
**

**
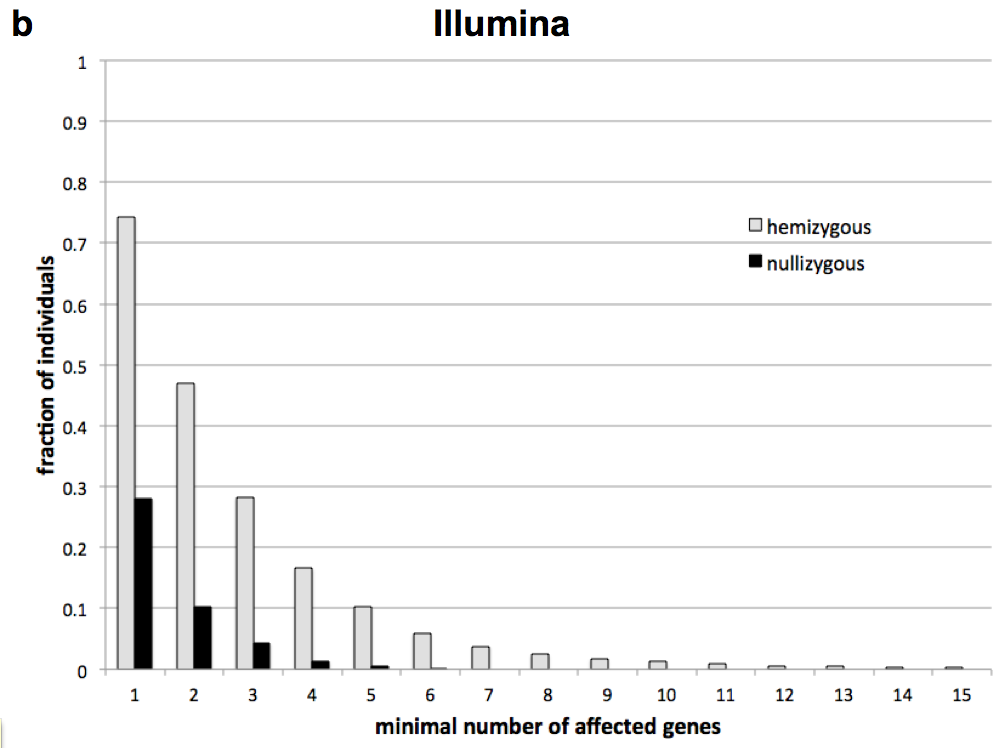
**

**Supplementary Table 1.** Commonly expected HMM states in the genome of the offspring, given the states observed in the parents (P1 and P2). For simplicity, we consider as “concordant” only combinations of deletions (states 0 and 1) and combinations of expansions (states 3 and 4), but not combinations of deletions and expansions. While such combinations are technically possible, they are rare; their inclusion would lead to significant loss of signal. Segments with unexpected combinations of states thus represent either such rare combinations of deletions and expansions, *de novo* CNV changes (also expected to be rare) or, more typically, segmentation errors.

| **P1 \ P2** | **0** | **1** | **2** | **3** | **4** |
| --- | --- | --- | --- | --- | --- |
| **0** | 0 | 0 or 1 | 1 |  |  |
| **1** | 0 or 1 | 0 or 1 or 2 | 1 or 2 |  |  |
| **2** | 1 | 1 or 2 | 2 | 2 or 3 | 3 |
| **3** |  |  | 2 or 3 | 2 or 3 or 4 | 3 or 4 |
| **4** |  |  | 3 | 3 or 4 | 4 |
